# From plan to practice: a structured report on implementation strategies for preventing non-ventilator hospital-acquired pneumonia (nvHAP)

**DOI:** 10.1017/ice.2026.10422

**Published:** 2026-05

**Authors:** Aline Wolfensberger, Mirjam Faes Hesse, Hugo Sax, Lauren Clack

**Affiliations:** 1 Institute for Implementation Science in Health Care, University of Zurich, Zurich, Switzerland; 2 Department of Infectious Diseases and Hospital Epidemiology, University Hospital Zurich and University of Zurich, Zurich, Switzerland; 3 Department of Public and Global Health, Epidemiology, Biostatistics and Prevention Institute, University of Zurich, Zurich, Switzerland; 4 Department of Infectious Diseases, Bern University Hospital and University of Bern, Bern, Switzerland

## Abstract

**Background:**

There is increasing evidence on the effectiveness of prevention bundles against non-ventilator hospital-acquired pneumonia (nvHAP), but detailed reports on their implementation are lacking. This study aims to describe and structure the implementation activities undertaken in a single-center multimodal intervention that achieved a 31% reduction in nvHAP incidence.

**Design:**

Longitudinal descriptive qualitative study.

**Setting:**

Nine medical and surgical departments of a Swiss university hospital.

**Participants:**

Healthcare professionals and implementation teams in study departments.

**Methods:**

We collected longitudinal data on implementation activities using (1) implementation activity logs, (2) drop-in interviews and observations, (3) “action plan meetings,” (4) focus groups, and (5) unstructured recall sessions among the project team. Data were deductively coded using the “Expert Recommendations for Implementing Change” taxonomy, specified using Proctor et al.’s “Recommendations for specifying and reporting implementation strategies” and mapped to the “Exploration, Preparation, Implementation, Sustainment” framework phases.

**Results:**

A total of 174 activities were undertaken. Activities varied by implementation phase, most frequently involving “evaluative and iterative strategies,” “develop stakeholder interrelationship strategies” and “training and education of stakeholders” during Exploration, Preparation, and Implementation, respectively. During Implementation, 54% of activities were initiated by department nurses, and 27% were initiated by the institutional implementation team. Activities included interdisciplinary kick-off events, education in various formats, posters, informational stickers for patients, provision of new equipment (e.g., toothbrushes), and electronic medical records order sets.

**Conclusions:**

This report offers valuable insights for future implementation efforts by providing a structured overview of the concrete implementation activities performed in a successful one-hospital multimodal nvHAP prevention project.

## Introduction

Hospital-acquired pneumonia (HAP) is one of the most common healthcare-associated infections (HAI).^1–3^ About one-third of cases are ventilator-associated pneumonia (VAP), occurring in intubated patients. The other two-thirds affect non-intubated patients, termed non-ventilator hospital-acquired pneumonia (nvHAP).^1,3^ NvHAP is associated with serious consequences, including longer hospital stays^4^ and increased mortality.^5,6^ With costs per episode estimated up to USD 28,000, it is also among the most expensive HAI.^7^ Despite its relevance, most guidelines and scientific articles focus on VAP, while nvHAP has only recently begun to receive attention as a “neglected HAI.”^8,9^

While evidence is growing for the effectiveness of individual nvHAP prevention measures and bundles,^10–12^ their implementation has been less studied. In a “call to action,” IPC experts emphasized that “it is not enough to know which prevention methods work under trial conditions; it is equally important to develop practical and generalizable strategies to help hospitals translate this evidence into real-world practice.”^9^ Publications on successful improvement programs can offer insights into implementing pneumonia prevention measures, but most focus on VAP, not nvHAP.^13^ Since nvHAP can affect all non-intubated patients across general wards, intermediate care, and intensive care units, the implementation context is more diverse, and effective strategies may differ from those used for VAP prevention.

A recent hybrid type-II effectiveness-implementation study at the University Hospital Zurich (USZ) evaluated a 5-element nvHAP prevention bundle across nine medical and surgical departments.^14,15^ Implementation led to a 31% reduction in nvHAP incidence.^14^ The quality improvement project and its scientific evaluation were led by the USZ infection prevention and control (IPC) team—physicians, nurses, and an implementation scientist. While earlier publications described the overarching multifaceted strategy,^14,15^ this paper provides a structured account of implementation activities undertaken by the institutional and the department implementation teams during each project phase.

## Methods

### The intervention

**Figure 1.**
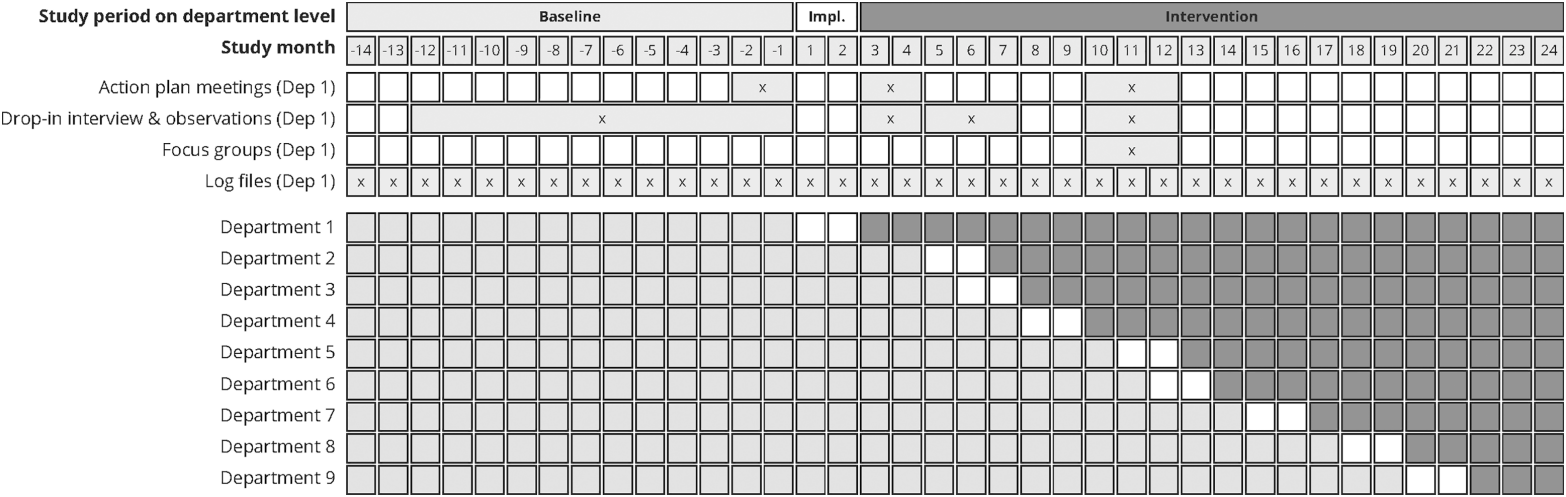
Study timetable including data collection timepoints. *Note*: Graphic representation of study periods (baseline, implementation, and intervention) and data collection timepoints for Department 1. Each box represents one study month, with Month 1 indicating the start of implementation in Department 1. Merged boxes in the data collection segment indicate that data was collected during this time period. Dep, Department; Impl, Implementation.

This structured description of nvHAP prevention implementation is based on qualitative data from a single-center, type-II hybrid effectiveness–implementation study conducted between 2017 and 2020 across nine surgical and medical departments of USZ, a 950-bed tertiary care center.^14,15^ The quasi-experimental study, with a non-randomized stepped-wedge design, included three department-level study periods: (1) a 14–31-months “baseline” period, (2) a 2-month “implementation” period initiated at each department’s discretion, and (3) a 3–22-month “intervention” period, shortened due to early termination during the COVID-19 pandemic. The total study duration was 38 months (Figure 1). The nvHAP prevention bundle included five measures: oral care, dysphagia screening and management, mobilization, discontinuation of non-indicated proton pump inhibitors (PPI), and respiratory therapy. Details on trial design, effectiveness, and implementation outcomes are reported elsewhere.^14,15^

### Data collection, analysis, and reporting

Prospectively collected data from the project were used for the present analysis. All implementation activities (hereafter, activities) were defined as specific, purposeful actions or tasks undertaken by the institutional implementation team (*InstTeam*) or department implementation teams (*DeptTeam*) to put the prevention measures into practice. Activities were assigned to one or more implementation *Strategy Cluster* and were counted once, even if repeated (e.g., identical educational sessions). Three researchers (MFH, LC, AW) documented activities using:

1. activity logs based on internal meetings and departments interactions (e.g., support calls, emails, or activity notifications);
2. unstructured field notes and observations during drop-in interviews; and
3. transcripts and unstructured notes from “action plan meetings,” focus groups, and internal *InstTeam* discussions.

Figure 1 outlines the data collection timepoints. Data for the early project stages (*Exploration* phase) were partially reconstructed retrospectively through collective recall sessions with the project team, all of whom are authors of this paper.

We used a structured approach guided by three frameworks to label and specify activities (Table 1). First, to categorize strategy type, we applied the nine implementation *Strategy Clusters* by Waltz et al.,^16^ which consolidate the original *Expert Recommendations for Implementing Change (ERIC)* (Table 2).^17^ Second, we used Proctor et al.’s framework to specify each activity’s actor (*InstTeam, DeptTeam,* or both) and target (nurses, physiotherapists, physicians, or patients).^18^ Third, we used the *Exploration, Preparation, Implementation, Sustainment (EPIS)* framework to capture the temporal sequence of implementation phases:^19^

**Table 1.** The four dimensions of implementation activities

Dimension	Explanation
**EPIS** framework phases (Exploration, Preparation, Implementation, Sustainment)^19^	***Exploration***, identifying needs and selecting a mitigation strategy; ***Preparation***, identifying barriers and facilitators, planning the implementation and its support and creating a favorable climate; ***Implementation***, initiating evidence-practice use; and ***Sustainment***, establishing structures, processes, and supports to secure continues practice delivery.
**Strategy Clusters** (Waltz et al.^16^ based on ERIC taxonomy)^17^	Grouping of implementation *Activities* into nine *Strategy Clusters* (Table 2) based on the Expert Recommendations for Implementing Change (ERIC) taxonomy of implementation strategies: ***Adapt and tailor to context*** ***Change infrastructure*** ***Develop stakeholder interrelationships*** ***Engage consumers*** ***Provide interactive assistance*** ***Support clinicians*** ***Train and educate stakeholders*** ***Utilize financial strategies*** ***Use evaluative and iterative strategies***
**Activity Roles** (Proctor et al.)^18^	The ***Actor***, the agent delivering the implementation strategy, either the Institutional implementation team (*InstTeam)* or Department team (*DeptTeam)* The ***Target*,** the recipient of the strategy, such as professional groups, nurses, physiotherapists, physicians, and patients
**Bundle Elements**	***Oral care*** ***Dysphagia screening and management*** ***Mobilization*** ***Stopping non-indicated proton pump inhibitors*** ***Respiratory therapy*** ***Bundle as a whole***

**Table 2.** Implementation activities per implementation strategy cluster and EPIS phase

	EPIS Phases			
Implementation Strategy Cluster	Exploration (n = 4 activities)	Preparation (n = 16 activities)	Implementation (n = 147 activities)	Sustainment (n = 54 activities)
Adapt and tailor to context	–	2 (13%)	5 (3%)	4 (7%)
Change infrastructure	–	3 (19%)	23 (16%)	24 (44%)
Develop stakeholder interrelationships	–	9 (56%)	28 (19%)	7 (13%)
Engage consumers (i.e. patients)	–	2 (13%)	13 (9%)	5 (9%)
Provide interactive assistance	–	1 (6%)	3 (2%)	3 (6%)
Support clinicians	–	–	21 (14%)	6 (11%)
Use evaluative and iterative strategies	4 (100%)	2 (13%)	8 (5%)	1 (2%)
Train and educate stakeholders	–	2 (13%)	109 (74%)	22 (41%)
Utilize financial strategies	–	2 (13%)	–	–

Due to early study termination, actual sustainment was not observed. However, we anticipated sustainment when activities were:

- embedded in electronic medical record (EMR)
- incorporated into standard operating procedures, or
- designated as recurring educational or training modules.

Three authors (MFH, AW, LC) deductively coded activities, resolving discrepancies by consensus.

The ethics committee of the Canton of Zurich, Switzerland, waived formal evaluation for this quality improvement project (Req-2017-00731). Written informed consent was obtained from healthcare practitioners (HCPs) participating in audio-recorded interviews.

## Results

The qualitative data set included 27 action plan interviews (three per department), 160 drop-in interviews with field notes, nine focus groups, ten log files (one per department and one InstTeam file), and notes from the recall sessions. Across all nine departments, four activities occurred during *Exploration*, 16 during *Preparation*, and 154 during *Implementation*. Of these, 54 were expected to support sustainability and were also attributed to the Sustainment phase. Activities by *Strategy Cluster* and EPIS phase are detailed in Table 2 and described below.

### Exploration

All four activities in *Exploration* were assigned to the “use evaluative and iterative strategies” strategy cluster and conducted by the *InstTeam* actor. They included two surveillance activities and two activities to assess the need for change.

Motivation arose from repeated hospital-wide point prevalence surveys showing pneumonia as the second most common HAI—less than half of which were VAP. In response, the USZ IPC team implemented a semi-automated nvHAP incidence surveillance in 2017, enabling the identification of departments with the highest nvHAP risk.^20^ At the time, the hospital lacked nvHAP prevention guidelines, and no national or international guidelines on nvHAP prevention could be found. A review of original literature identified potentially effective and safe prevention measures, highlighting oral care as a key intervention.^21^ Interviews by an IPC nurse with frontline HCP revealed limited awareness of oral care’s preventive role and its low prioritization in daily practice. Additional, scoping interviews uncovered poor adherence to other preventive measures, such as patient mobilization.

In 2016, the hospital had launched a broader IPC campaign—the ”5% Offensive”—aiming to reduce HAI point prevalence from >8% to below 5%, through surveillance and monitoring of prevention measures of all major five HAI. By 2017, the IPC team began preparing a dedicated nvHAP prevention project.

### Preparation

During *Preparation*, 16 activities were carried out across eight clusters by the *InstTeam* as sole actor, most frequently in the “develop stakeholder interrelationship” cluster (9 of 16; 56%).

The *InstTeam—*comprising an IPC physician (AW), IPC nurse (MTM), and an implementation scientist (LC)—was established to lead the project preparation. In collaboration with an interprofessional expert group (nurses and physiotherapists), and informed by the literature, the team developed an institutional nvHAP prevention guideline. It was approved by the IPC board and hospital leadership and published on the hospital intranet.

The head of IPC (HS) and the *InstTeam* secured project support from the hospital directorate, positioning it as part of the institutional “5%-Offensive.” Funding was obtained from the Swiss Federal Office of Public Health under the national Strategy NOSO,^22^ enabling the recruitment of a study nurse (MFH), who joined the *InstTeam* and served as the primary contact for *DeptTeams*.

Based on surveillance data, nine departments with above-average nvHAP incidence were selected. The *InstTeam* held meetings with the hospital leadership and department delegates to obtain participation consent. *DeptTeams* were formed in each department, typically including a nurse, a physician, and a physiotherapist, appointed by their respective medical and nursing directors. This approach aimed to foster local ownership by tailoring the activities to department needs.

Drawing on insights from *Exploration*, the *InstTeam* defined the three core implementation strategies,—“education,” “training,” and “environmental restructuring”—alongside additional context-specific strategies. An initial plan for random assignment of implementation start dates for each department was later abandoned for feasibility reasons.

The *InstTeam* facilitated implementation through regular “action plan meetings” with *DeptTeams* to share barriers, facilitators, and lessons learned, supporting formative evaluation and ongoing adaptation.^23^ Additional *InstTeam* activities included engaging a graphic designer for educational materials and collaborating with the EMR team to improve documentation and prescribing of nvHAP prevention measures. Surveillance of nvHAP continued throughout this phase.

### Implementation

During *Implementation*, activities were carried out by *DeptTeams*, the *InstTeam*, or both, tailored to local needs. When formative evaluation revealed shared challenges, the *InstTeam* introduced department-spanning strategies—eight in total—across all nine participating departments. Two were educational in nature: a five-minute instructional video for nurses on the indications for and conduct of dysphagia screening, published on the hospital intranet; and a leaflet for the patient hospitality service addressing preventive aspects of patient mobilization. Two served as clinician reminders: posters outlining nvHAP prevention measures and indications for respiratory therapy, displayed in physicians’ offices and ward staff rooms; and an indication list for proton pump inhibitors, developed in collaboration with a gastroenterologist and made accessible via the intranet. Then, patients were addressed through a sticker mounted on bathroom mirrors highlighting preventive aspects of oral care. In addition, a new oral hygiene kit—including a high-quality, colorful, soft toothbrush and an informational leaflet—was introduced hospital-wide. *DeptTeams* received semiannual feedback on adherence rates and nvHAP surveillance results from the infection prevention team. Finally, action-plan meetings between *DeptTeams* and the *InstTeam* were conducted on an ongoing basis.

**Figure 2.**
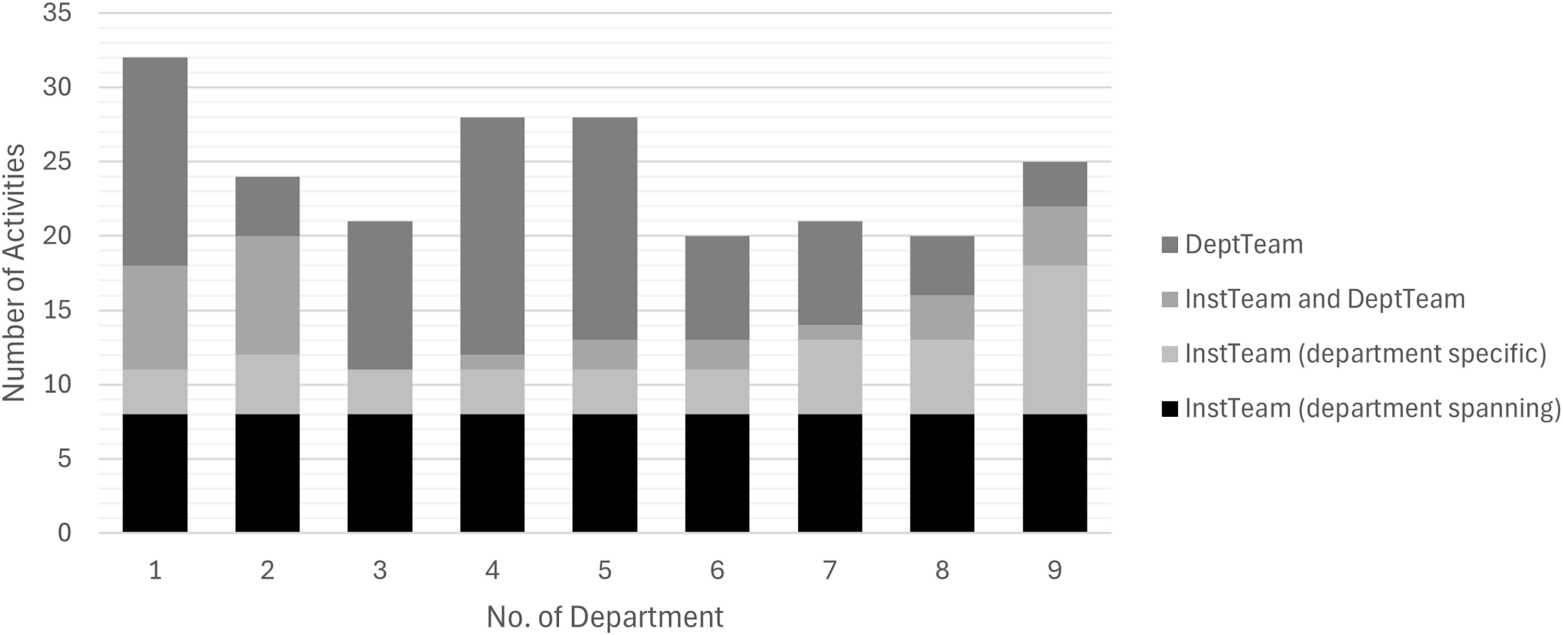
Activities by actors per department during Implementation phase. *Note*: Number of *Activities* taken by the department team (*DeptTeam*), the institutional implementation team (*InstTeam*) or both (*InstTeam* and *DeptTeam*). Department-specific activities were defined as delivered only in one department, department-spanning activities across all nine departments. An activity is a distinct, concrete action or method in a department executed once or repetitively, carried out with the goal to implement one or several nvHAP prevention measures. No, number.

In addition to these department-spanning activities, 147 department-specific activities were performed across the nine departments, 12 to 24 per department (Figure 2). *DeptTeams* carried out just over half (n = 80, 54%), while the *InstTeam* conducted 39 (27%) upon department request. The remaining 28 (19%; range: 0%–50%) were joint efforts. Only in department 9 did the *InstTeam* contribute more activities than the *DeptTeam*.

In department-specific activities, nurses were most frequently both implementation actors and targets. Physicians were common targets but rarely actors (Table 3). Physiotherapists and patients were infrequent targets. Most activities concerned the overall bundle, followed by “oral care” and “dysphagia screening,” and limited focus was on “respiratory physiotherapy” or “stopping non-indicated PPI.”

**Table 3.** Implementation actor and implementation target of department-specific activities per bundle element

	Oral Care (n = 35 activities)	Mobilization (n = 9 activities)	Dysphagia screening & Management (n = 24 activities)	Stopping PPI (n = 5 activities)	Respiratory Physio (n = 5 activities)	nvHAP bundle (n = 74 activities)
**Implementation actors**						
Nurses*	27	4	20	2	–	29
Physicians*	–	1	1	3	1	3
Physiotherapists*	–	3	3	–	1	5
Mixed professional groups (of the three mentioned above)*	–	–	–	–	2	8
Patients	–	–	–	–	–	–
Institutional Implementation Team only	4	–	1	–	1	33
**Implementation targets**						
Nurses	31	5	21	2	–	38
Physicians	–	2	2	3	3	14
Physiotherapists	–	2	1	–	–	5
Mixed professional groups (of the three mentioned above)	–	–	–	–	2	16
Patients	4	1	–	–	–	5

Numbers represent the absolute number of activities carried out per bundle element and actor/target group. Implementation actors carried out the implementation activities, while implementation targets received them. Mixed groups typically included representatives from all three professions but occasionally comprised only two professional groups. NvHAP bundle was coded if 3 or more bundle elements were subject of the implementation activity. Darker shade of grey represents higher frequency of activities applied per targeted bundle element.

* Activities performed by nurses, physicians, and physiotherapists were either supported or unsupported by the institutional implementation team.

Table 4 summarized strategy clusters and activities used per bundle element. “Train and educate stakeholders” was by far the most used cluster. Education relied mostly on short-format (5–15 minutes) sessions for a small number of participants, including ward-based inputs, speed learnings, and brief lectures. These sessions were often repeated to maximize penetration. These brief educational sessions were supported by longer (up to 60 minutes) educational sessions for larger groups, often including practical exercises or in-depth discussion. The most common topics were oral care, mobilization, dysphagia screening or nvHAP prevention in general. In many of these sessions, specialist experts (e.g., dentists, physiotherapists) were deliberately integrated where (technical) expertise was essential, strengthening credibility and quality of sessions.

**Table 4.** Implementation activities per implementation strategy cluster and bundle element

Strategy Clusters (Waltz et al)	Oral Care	Mobilization	Dysphagia Screening and Management	Stopping non-indicated PPI	Respiratory Physiotherapy	nvHAP bundle
Adapt and tailor to context	0	0	4	0	0	1
Change infrastructure (Core strategy)	11	2	5	0	0	5
Develop stakeholder interrelationships	2	1	2	0	2	21
Engage consumers (i.e. patients)	7	1	0	0	0	5
Provide interactive assistance	1	1	0	0	0	1
Support clinicians	2	1	2	1	1	14
Use evaluative and iterative strategies	5	0	1	0	0	2
Train and educate stakeholders (Core strategy)	13	11	20	5	4	56

Numbers represent the absolute number of activities carried out per strategy cluster and bundle element, with NvHAP bundle defined as 3 or more bundle elements were subject of the activity. Darker shade of grey represents higher frequency of strategy cluster applied or targeted bundle element.

Strategies to develop and strengthen stakeholder interrelationships focused on establishing interdisciplinary collaboration, primarily among nursing staff, physicians, and therapists. The project was initiated through formal interprofessional kick-off events in two departments. In addition, several departments integrated nvHAP prevention aspects of patient care into existing interprofessional meeting structures. For specific bundle elements, such as oral care and mobilization, specialist expertise was actively sought, including consultation with dentists and therapists, which frequently resulted in the targeted educational sessions delivered to clinical teams mentioned above.

Infrastructure-related implementation strategies included the systematic integration of nvHAP bundle elements into the electronic medical record. This comprised the creation and refinement of standardized admission order sets for nursing staff, including oral care or dysphagia screening. Already existing documentation fields, e.g., for mouth status assessment were refined to improve usability. Beyond digital infrastructure, physical infrastructure was changed on certain departments: one department introduced suction toothbrushes, while another benefited from an already existing therapy room at the ward to conveniently perform physiotherapy.

Strategies to support clinicians primarily consisted of extensive use of reminders and practical decision-support tools. Posters, flyers, and checklists addressing nvHAP bundle elements, respiratory therapy indications, and PPI use were widely and strategically placed in wards, staff rooms, physicians’ offices, and on ward-round carts. In addition, laminated summaries and checklists were distributed to support day-to-day clinical decision-making. Some departments also expanded the competency of nurses to prescribe oral care products or to order a fascio-oral tract therapy for patients with suspected dysphagia.

In addition to the above-mentioned informational oral care stickers, patient engagement activities were implemented in some departments. One surgical department informed its patients through preoperative appointment materials, including an information leaflet on nvHAP prevention measures. Another department piloted patient information using folded cards and plastic cubes; however, this initiative was discontinued due to feasibility issues. Several departments actively sought direct patient feedback on the visibility and usefulness of patient information materials, and these findings informed subsequent adaptation and scaling of materials across departments.

**Figure 3.**
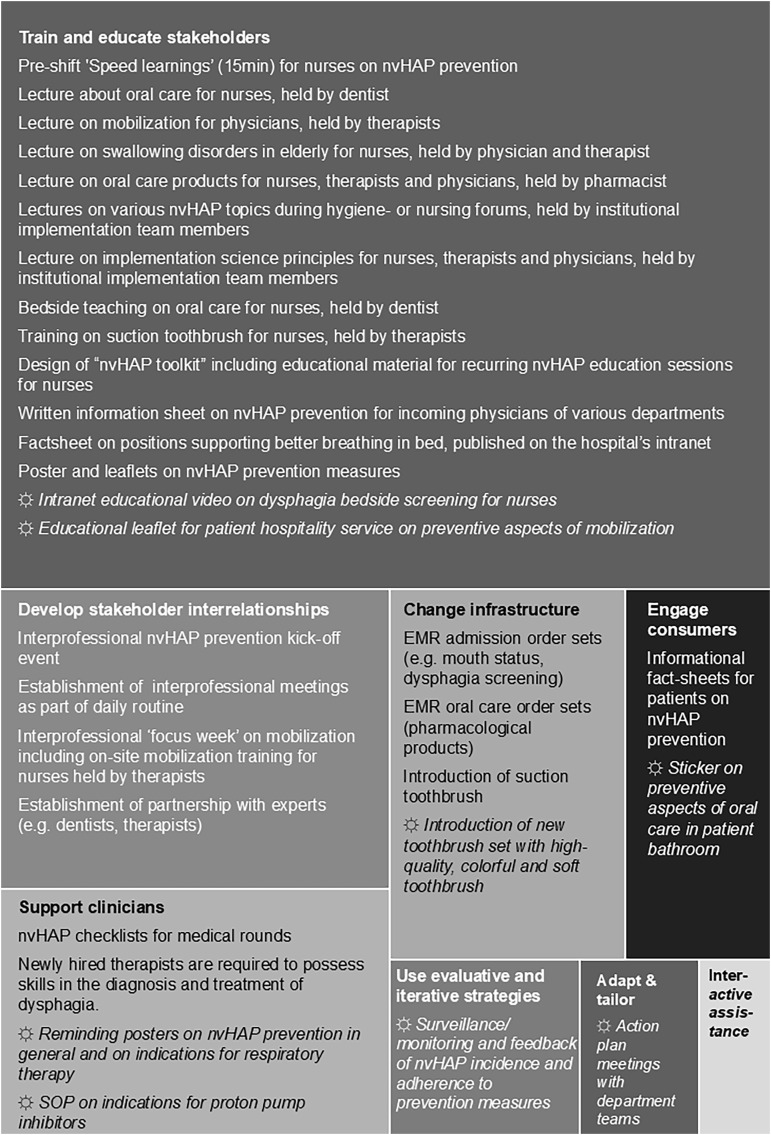
Treemap diagram with examples of implementation activities per strategy clusters. *Note*: Examples of implementation activities for the eight applied implementation strategy clusters (boxes of the treemap diagram), displayed as department-specific activities (normal font) and department-spanning activities (☼ and *italic font*). One strategy cluster (Utilize financial strategies) was not used at all. EMR, electronic medical record; eval./iterat., evaluative / iterative; Interact. ass., Provide interactive assistance; nvHAP, non-ventilator hospital-acquired pneumonia; prev., prevention; SOP, standard operating procedure.

Figure 3 provides a graphical overview of the frequency of applied strategy clusters alongside example activities.

### Sustainment

Of the activities carried out during the *Implementation* phase, 54 also addressed *Sustainment*. In 48% (n = 26) the *InstTeam* was the *Actor*. Many (n = 24) fell under the “Change infrastructure” strategy cluster, mainly involving changes in the electronic medical record defaults, such as those mentioned above. Additionally, 22 “Train and educate stakeholders” strategy cluster activities*—*if including repetitive or continuous provision of nvHAP educational materials (e.g., PPI-indication list, educational video about swallowing screening), were also assigned to *Sustainment*.

## Discussion

This paper provides insights into the actual implementation activities of a successful nvHAP prevention project. We detail institutional and department level *a*ctivities—along with their actors and targets*—*for implementing nvHAP bundle elements across the four EPIS phases in a Swiss university hospital. During *Exploration*, “Evaluative and iterative” activities identified nvHAP prevention as a relevant patient need and led to launching a comprehensive theory-based, scientifically evaluated quality improvement project, supported by institutional and national funding. In *Preparation*, activities revolved around building partnerships, establishing hospital guidelines, and defining core implementation strategies. During *Implementation*, *DeptTeams* tailored implementation activities to local needs, performing half of them independently while relying on support from *InstTeam* for the other half. In *Implementation* and *Sustainment*, activities heavily relied on “Education and training of stakeholders.”

Although the multifaceted implementation strategy, including three core strategies, was “designed to increase ownership and local adoption” by engaging *DeptTeams,* ^15^ the *InstTeam* remained the sole actor for eight department-spanning activities. These were initiated in response to needs identified through formative evaluation. Similarly, the *InstTeam* frequently executed department-specific activities—either independently or in collaboration with *DeptTeams—*based on department requests. Data from action plan meetings and focus groups suggested that departments might have independently developed similar strategies, but the central support was time- and resource-efficient and therefore an often cited key facilitator. The *InstTeam’s* active involvement exceeded initial plans. Originally, it aimed to enable a primarily department-led implementation through facilitation and nudging. However, high clinical workloads and limited implementation expertise likely impeded this, resulting in a more externally driven process—though still internal to the hospital.^24^

In the *DeptTeams*, the nurses were the most active professional group, with most implementation efforts focused on “oral care” and “dysphagia screening and management.” This reflects the nursing focus of several bundle elements and aligns with findings from several postoperative pneumonia prevention projects, where activities primarily targeted nurses.^12,25,26^ Physiotherapists were the target of fewer activities, likely due to high baseline fidelity, perceived appropriateness and acceptability of relevant measures.^14^ The physiotherapists’ existing knowledge, skills, and motivation regarding “mobilization,” “dysphagia management,” and “respiratory therapy” also contributed. Physician engagement was comparatively low, possibly because “stopping non-indicated PPI” was the only bundle element directed specifically at them. Additional barriers may have included high clinical workload, a therapeutic rather than preventive focus, or lower acceptability and perceived appropriateness of the bundle among physicians.^14^ While physicians rarely led activities, they often participated in interprofessional activities—essential for a bundle requiring coordinated action across all professional groups.

During *Implementation*, the nine departmental teams selected between 12 and 24 activities to implement the nvHAP prevention bundle within their respective departments. Most activities focused on “Education and training.” This is unsurprising for two reasons: first, these were core strategies recommended by the *InstTeam* based on knowledge and skill gaps identified in the *Exploration* phase. Second, education is one of the most used implementation strategies in healthcare quality improvement. For example, Trogrlic et al. found all 21 ICU delirium intervention studies included education,^27^ and Tomasone et al. reported its use in 24 of 33 cancer care studies.^28^ While education and training have been critiqued as low-impact,^29^ and non-sustainable implementation strategies, they were essential to address gaps in frontline staff knowledge and skills—particularly for oral care and dysphagia screening.^30^ We agree, however, that one-off training is insufficient, and repetition is needed to support sustained behavior change. Several of the educational strategies selected by the *DeptTeams* were repetitive and thus fulfilled sustainment requirements.

This study has several limitations. First, it was conducted in a single tertiary care hospital in a high-income country, limiting generalisability of our findings. Second, this analysis relied on indirect sources such as action plan meetings and field notes, which might have missed details such as participants numbers or session durations. Third, while tailoring to local context was encouraged, departmental context adaptations were not systematically documented, limiting evaluation of this aspect. Finally, *InstTeam* members were both implementers and evaluators, introducing potential for desirability bias. To mitigate this, we followed a structured, prespecified evaluation protocol, used triangulated data from multiple sources, and engaged in regular reflexive discussions.

In conclusion, this study offers a detailed account of activities, strategies, and actors across all phases of a successful nvHAP prevention bundle implementation in a tertiary-care hospital. By outlining both institutional and departmental efforts, it offers practical guidance for future implementations. Further data analysis will explore potential causal links between specific strategies and outcomes. As others have noted,^9^ prospective and comparative trials are needed to identify the most effective implementation strategies for preventing nvHAP and other HAI.
